# Analogs of the Prime Number Problem in a Shot Noise Suppression of the Soft-Reset Process

**DOI:** 10.3390/nano15171297

**Published:** 2025-08-22

**Authors:** Yutaka Hirose

**Affiliations:** Panasonic Industry Co., Ltd., Kadoma City 571-8506, Osaka, Japan; hirose.yutaka@jp.panasonic.com

**Keywords:** CMOS image sensor, soft-reset, kTC noise, shot noise, hypoexponential distribution function, cumulants, Kolmogorov–Bateman equation, Markov chain, single-charge Coulomb energy, prime numbers, zeta function

## Abstract

The soft-reset process, or a sequence of charge emissions from a floating storage node through a transistor biased in a subthreshold bias condition, is modeled by a master (Kolmogorov–Bateman) equation. The Coulomb interaction energy after each one-charge emission leads to a stepwise potential increase, giving correlated emission rates represented by Boltzmann factors. The governing probability distribution function is a hypoexponential type, and its cumulants describe characteristics of the single-charge Coulomb interaction at room temperature on a mesoscopic scale. The cumulants are further extended into a complex domain. Starting from three fundamental assumptions, i.e., the generation of non-degenerated states due to single-charge Coulomb energy, the Markovian property of each emission event, and the independence of each state, a moment function is identified as a product of mutually prime elements (algebraically termed as prime ideals) comprising the eigenvalues or the lifetimes of the emission states. Then, the algebraic structure of the moment function is found to be highly analogous to that of an integer uniquely factored into prime numbers. Treating the lifetimes as analogs of the prime numbers, two types of zeta functions are constructed. Standard analyses of the zeta functions analogous to the prime number problem or the Riemann Hypothesis are performed. For the zeta functions, the analyticity and poles are specified, and the functional equations are derived. Also, the zeta functions are found to be equivalent to the analytic extension of the cumulants. Finally, between the number of emitted charges and the lifetime, a logarithmic relation analogous to the prime number theorem is derived.

## 1. Introduction

Shot noise is an electromagnetic fluctuation phenomenon due to random transports of individual charges across a potential barrier [1]. Reflecting such discrete and random nature, the phenomenon is commonly modeled as a train of random pulses [2,3]. Classically, under assumptions of stationarity (constant rate of the charge emission events) and of Markovian property (memoryless and therefore independence of each state), the governing probability distribution function (PDF) is derived as the Poisson distribution [2,3]. With the recent development of image sensors with the soft-reset (SR) schemes, where a charge storage node is reset by a reset transistor (RST) biased in a subthreshold regime with the emission rate inversely proportional to an exponential of the potential between the source and the gate of the RST [4,5,6,7,8,9,10], of quantum/mesoscopic devices [11,12,13,14], and of avalanche photodiodes [15], shot noise deviating from the stationary Poisson distribution has been recognized. In many cases, the origin of the phenomenon is attributed to the Coulomb charge correlation effect. To investigate such non-stationary shot noise, the full counting statistics (FCS) method based on analyses of high-order cumulants is developed [11,12,13,14,15]. As a special case, an analytical solution of a master (Kolmogorov–Bateman (KB)) equation giving the FCS of the shot noise suppression process during the SR was reported [16,17]. There, a floating charge storage node reset by a transistor biased in a subthreshold regime rendered a correlated potential transition with an equal step height after each single carrier emission (also called a feedback effect [5,16]). The governing PDF was found to be a hypoexponential distribution function (HDF). The cumulants are sums of converging geometric series with the common ratios composed of Boltzmann factors. Thus, the analytic forms of cumulants of any order could be derived. They describe the shot noise suppression due to the successive single-charge correlation effect, according to the reported experimental results [17]. The significance of the results is that the effect is observable at room temperature in mesoscopic-scale devices. Although shot noise suppression due to a sequence of single-charge transports was formulated as an HDF with a single electron transistor (SET) network [18], it is presumably operated at a cryogenic temperature. In this paper, we extend the cumulants into a complex domain to further exploit the mathematical characteristics of the single-charge emission events. Starting from three fundamental assumptions, i.e., (i) a potential transition with an equal step height corresponding to single-charge Coulomb energy; (ii) the Markovian (memoryless) property of each emission event; and (iii) the independence of each state, an equivalent circuit model of the SR-process is presented. Then, these assumptions are shown to lead to the characteristic algebraic structures of the PDF and of its cumulants, highly analogous to those of the integer ring [19]. In particular, the special role of the lifetime (inverse of the emission rate) of each state is identified as the prime element (algebraically termed as “prime ideals”) of the moment function analogous to the prime numbers producing an integer. Then, associated zeta functions are constructed, and their analyticities and functional equations are investigated in a similar way to the prime number problem or the Riemann Hypothesis [20,21]. Finally, between the emission numbers and the lifetime, a logarithmic relation analogous to the prime number theorem (PNT) [19] is derived.

## 2. Summary of Derivation of the Probability Distribution Function, Cumulants, and Basic Assumptions

In this section, we summarize the basic methods of modeling the soft-reset process and derive the probability distribution function and the cumulants [16,17]. These intrinsic characteristics of the soft-reset process are extracted in order to separate the soft-reset noise from other possible noise sources such as a leakage current.

### 2.1. Methods of Modeling and Derivation of the Probability Distribution Function and the Cumulants

**Figure 1:**
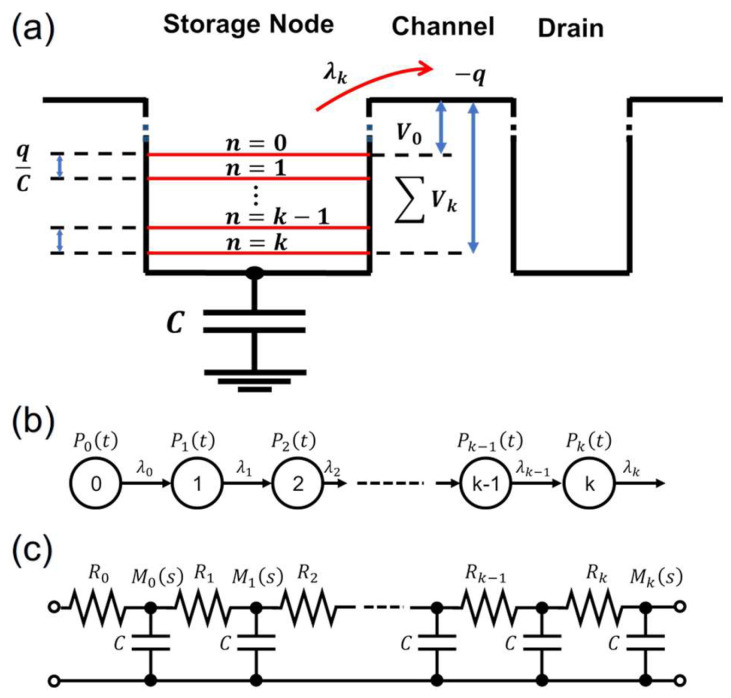
(**a**) A potential diagram of an SR process. The floating voltage of the storage node increases after each electron emission by *q*/*C*. (**b**) A state transition diagram. (**c**) An equivalent RC-ladder circuit model of the SR process.

A potential diagram is illustrated in Figure 1a. Discrete electrons on a floating diffusion storage node (hereafter, called FD) are sequentially emitted over a potential barrier set by a gate voltage of an RST. After each electron emission, the voltage of the FD increases by an amount of q/C where q is the unit of charge and C is the capacitance of the FD. The emission barrier voltage after the k-th electron emission (regarded as the FD in the k-th state) is

(1) $$V_{k}=V_{0}+\frac{kq}{C},$$

where $V_{0}$ is an initial barrier voltage. The RST is biased in a subthreshold regime with an emission rate in the k-th state as

(2) $$\lambda_{k}=\lambda_{0}\cdot exp\left(-\frac{kq^{2}}{k_{B}TC}\right),$$

where $\lambda_{0}$ is the initial emission rate. To realize the soft-reset condition, it is crucial to have a condition that $\left|qV_{0}\right|\gg k_{B}T\gg q^{2}/C$. The first inequality imposes a (subthreshold) bias condition of the reset transistor where current is entirely due to thermionic emissions of charges. For example, at room temperature (RT), with sub-micron Si CMOS devices, $V_{0}$ should be higher than ~0.3 V, corresponding to slightly lower than typical PN-junction threshold voltages. The second inequality requires a sufficient number of emitted charges, resulting in an asymptotic regime where convergence of cumulants is allowed (see Equation (11) below). At RT, the condition leads to the capacitance value of 1 fF~5 fF or larger. These are realized in typical submicron (0.1 mm~0.5 mm) Si CMOS image sensors as a total capacitance of a reset transistor gate, a storage node, and a parasitic capacitance. Thus, the soft-reset conditions are inherently related to mesoscopic-scale devices. On the other hand, at a very low temperature or with very small scale-devices such as quantum dots, the conditions would break down. These aspects will be briefly discussed in Section 4.2. In order to guarantee the soft-reset conditions, in practical image sensors, the reset transistors are operated by a fast turn-on followed by a slowly and linearly switching-off pulse with a typical tail period of ~1 μs, so-called “tapered-reset” [8,10].

The master equation, or the KB equation, is set up as follows:

(3) $$\frac{d}{dt}P_{k}\left(t\right)\;=\lambda_{k-1}\cdot P_{k-1}\left(t\right)-\lambda_{k}\cdot P_{k}\left(t\right),$$

(4) $$\frac{d}{dt}P_{0}\left(t\right)=-\lambda_{0}\cdot P_{0}\left(t\right),$$

where $P_{k}\left(t\right)$ is a probability distribution function (PDF) that, at time t, the FD is in the k-th state. The initial conditions are; $P_{0}\left(0\right)\;=\;1$ and $P_{k}\left(t\right)\;=0\;(k\neq 0)$. Equations (3) and (4) are represented in a matrix form as follows:

(5) $$\frac{d}{dt}\vec{P}\left(t\right)\;=\;A\vec{P}\left(t\right),$$

where $\vec{P}\left(t\right)$ is a column vector

(6) $$\vec{P}\left(t\right)={\left[P_{0}\left(t\right),\;P_{1}\left(t\right),\;\ldots ,\;P_{k}\left(t\right)\right]}^{T},$$

and the *i*-th-row- and the *j*-th-column components of the matrix operator A are $A_{i,i}=\;-\lambda_{i}$, $A_{i,i-1}=\lambda_{i-1}$, and $A_{i,j}=\;0(j\neq i,\;i-1)$. It is noted that $A_{i,i}=-\lambda_{i}$ is the eigenvalue of A [16].

The solution of Equation (5) is $\vec{P}\left(t\right)\;=\exp\left(At\right)\cdot \vec{P}\left(0\right).\;$ To calculate exp(At), diagonalization of A is performed by solving the eigenvalue equation. The final form is a hypoexponential type probability distribution function [16],

(7) $$P_{k}\left(t\right)=\sum_{i=0}^{k}e^{-\lambda_{i}t}\cdot \frac{\prod_{j=0}^{k-1}\lambda_{j}}{\prod_{\begin{array}{c} j=0\\ j\neq i \end{array}}^{k}\left(\lambda_{j}-\lambda_{i}\right)}.$$

From Equation (7), the probability density function is derived as

(8) $$p_{k}\left(t\right)=\sum_{i=0}^{k-1}e^{-\lambda_{i}t}\cdot \prod_{\begin{array}{c} j=0\\ j\neq i \end{array}}^{k-1}\frac{\lambda_{j}}{\lambda_{j}-\lambda_{i}}.$$

It is noted that the notations are slightly modified from those in [16] for later use in the present work. Then, the k-th state moment generating function (MGF), $M_{k}\left(s\right)$, is obtained by Laplace transforming Equation (8),

(9) $$M_{k}\left(s\right)=\int_{0}^{+\infty }p_{k}\left(t\right)\cdot e^{-st}dt=\prod_{j=0}^{k-1}\frac{\lambda_{j}}{\lambda_{j}+s}.$$

By differentiating m-times, the m-th order cumulant of the time variable, c(m, k), is

(10) $$c\left(m,\;k\right)\;\equiv {{\left(-1\right)}^{m}\left.\frac{d^{m}}{ds^{m}}\ln\left(M_{k}\left(s\right)\right)\right|}_{s=0}={{\left(-1\right)}^{m+l}\left.\frac{d^{m-l}}{ds^{m-l}}\sum_{i=0}^{k-1}\frac{1}{{\left(\lambda_{i}+s\right)}^{l}}\right|}_{s=0}=\left(m-1\right)!\sum_{i=0}^{k-1}\frac{1}{{\lambda_{i}}^{m}},$$

where l is an integer satisfying l≤m. It is noted that the term associated with the numerator of the last term of Equation (9), $\prod_{j=0}^{k-1}\lambda_{j}$, is constant and reduced to zero after differentiation by s. The m-th order, k-th state cumulant represented by the number of emitted charges, $Cum\left(m,\;k\right)$, is obtained by multiplying ${\lambda_{k-1}}^{m}$ to each term in the summation of Equation (10) as

(11) $$Cum\left(m,\;k\right)\;\equiv \;{\lambda_{k-1}}^{m}\cdot c\left(m,\;k\right)=\left(m-1\right)!\sum_{i=0}^{k-1}{\lambda_{i}}^{m}=\left(m-1\right)!\cdot \frac{1-\exp\left(-\frac{k\cdot mq^{2}}{k_{B}T\cdot C}\right)}{1-\exp\left(-\frac{mq^{2}}{k_{B}T\cdot C}\right)}.$$

The key point is the second equality, after which the common ratio of the geometrical series is converted to less than unity, giving the final result as a finite sum even in the limit of k→∞.

The soft-reset noise or $Cum\left(2,\;\infty \right)\;=\;\frac{1}{2}\frac{k_{B}T\cdot C}{q^{2}}$ is a well-established result obtained in the image sensor laboratories over the last quarter of a century. In particular, the origin of the pre-factor $\frac{1}{2}$ was a big mystery. The situation is referred to in references [4,5,6,7,8,9,10]. Equations (1)–(6) are confirmed to be correct when used in step-by-step simulations for $Cum\left(2,\;\infty \right)$ in references [5,9]. However, its analytical solution was not available until references [15,16]. In [15,16], the analytical solutions of Equations (1)–(6) are confirmed to be correct for $Cum\left(2,\;\infty \right).$ In addition, as for the pre-factor, it was not only correctly obtained but also its denominator is described as a part of a regular integer series without any adjusting parameter.

### 2.2. Essential Assumptions and an Equivalent Circuit Model

Fundamental assumptions in the formulation from (1) to (4) are: (i) the single-charge potential transition after each emission due to the Coulomb correlation (feedback effect [5,16]; (ii) the Markovian (memoryless) property of each emission event; and (iii) the independence of each electron emission state. Assumptions (i) and (ii) establish nondegeneracy of the states, making the state and the emitted number of charges sequentially countable as illustrated in Figure 1a (potential diagram) and in Figure 1b (state transition diagram). Assumption (ii) also gives each (i-th) state an exponential decaying probability function of which MGF has a form of $1/\left(\lambda_{i}+s\right)$. Then, combined with assumption (iii), the total MGF is obtained as the product of each state’s MGF as Equation (9). The MGF of each state is equivalent to a transfer function of a series R-C circuit with the connection point of R and C being the output node. The time constant (lifetime) of the R-C circuit is $1/\lambda_{i}$. Thus, the whole sequence may be described by an R-C ladder circuit as illustrated in Figure 1c. The resistance value of the *i*-th state, $R_{i}$, is obtained by equating the above two time constants, ($R_{i}C\;=1/\lambda_{i}$) as

(12) $$R_{i}=\frac{1}{C}\cdot exp\left(\frac{i\cdot q^{2}}{k_{B}TC}\right).$$

Equation (12) represents an ohmic resistance with a single-charge emission voltage, q/C, and an average current of the *i*-th state, ${q\lambda }_{i}$. It is noted that with assumptions (ii) and (iii), one obtains a common stationary shot noise described by the Poisson distribution [2,3]. Thus, the addition of assumption (i) should produce the distinguished characteristics as explained below.

## 3. Results

In the following, we will show how the above three assumptions lead to the mathematical structure of the SR-process being highly analogous to prime numbers forming integers.

### 3.1. Lifetime of a State as a Prime Element

Assumption (iii) leads to the MGF comprising a product of algebraically “mutually prime” terms (termed as prime ideals [22]), i.e., each state’s MGF derived from assumptions (i) and (ii). Its general form is obtained as

(13) $$G_{k}\left(s\right)=\alpha \prod_{i=0}^{k-1}{\left(\frac{1}{\lambda_{i}+s}\right)}^{n_{i}},$$

where α is a real constant and $n_{i}$ is a repetition factor of i-th state. Evaluating (13) at s = 0, we obtain

(14) $$G_{k}\left(0\right)=\alpha \prod_{i=0}^{k-1}{\left(\frac{1}{\lambda_{i}}\right)}^{n_{i}}=\;\alpha \prod_{i=0}^{k-1}{\tau_{i}}^{n_{i}},$$

where $\tau_{i}$ is the lifetime of the *i*-th state defined as

(15) $$\tau_{i}\equiv 1/\lambda_{i}.$$

Equation (14) may be regarded as a generalized moment factored into a unique product of $\tau_{i}$. The algebraic structure of Equation (14) is highly analogous to that of the integer ring; an integer number, N, factored into a unique product of prime numbers, $p_{i}$:

(16) $$N=\prod_{i=1}^{k}{p_{i}}^{n_{i}}.$$

Thus, we may regard $\tau_{i}$ as a “prime element” generating the generalized moment. Formal discussions on correspondence between polynomial rings and the integer ring are made in algebra texts [22]. Then, the analytic properties of Equation (14) can be analyzed in a similar way to that developed in the prime number problem or the Riemann Hypothesis: (i) constructions of the zeta functions, (ii) investigations of their analyticity, and (iii) their functional equations [19,20,21].

### 3.2. Analyses of a Linear Operator Zeta Function

From eigenvalues of a linear operator, a zeta function is constructed as follows [23]:

(17) $$\zeta_{lo}\left(s\right)\;=\;\sum_{i}{\lambda_{i}}^{s}\;=\;\sum_{i}{\tau_{i}}^{-s},$$

where s is a complex variable. $\zeta_{lo}\left(s\right)$ is derived from a more fundamental Dirichlet series [24] defined as

(18) $$\sum_{i}a_{i}\exp\left(-\theta_{i}s\right).$$

By setting $a_{i}\;=\;1$ and $\theta_{i}\;=\;ln(\tau_{i})$, Equation (17) is obtained. If we set $a_{i}\;=\;1$ and $\theta_{i}\;=\;ln(i)$, we obtain the Riemann zeta function for the integers as

(19) $$\zeta_{R}\left(s\right)=\sum_{i}i^{-s}=\frac{1}{1^{s}}+\frac{1}{2^{s}}+\frac{1}{3^{s}}+\ldots .$$

In general, this constructed zeta function is analytic everywhere except for poles [23]. In the present case, since the explicit form of each $\lambda_{i}$ is known as Equation (2), it is possible to analyze Equation (17) explicitly. Using the sum formula of geometric series and setting $\lambda_{0}\;=\;1$ for simplicity,

(20) $$\zeta_{lo}\left(s\right)=\sum_{i}\exp\left(-i\cdot sa\right)=\frac{1}{1-\exp\left(-sa\right)},$$

where

(21) $$a\equiv q^{2}/(k_{B}TC).$$

The second equality of (20) holds for $\left|\exp\left(-sa\right)\right|<1$. But since the third term of (20) is analytic except for poles, the function Equation (17) is analytically extended as Equation (20) to the entire complex plane except for poles.

Expressing s by complex plane coordinates,

(22) s=x+j·y,

where j is the imaginary unit, the poles of $\zeta_{lo}\left(s\right)$ are obtained by setting the denominator of Equation (20) to be zero.

(23) $$x=0,\;y=2n\pi \cdot \left(\frac{k_{B}TC}{q^{2}}\right),\;(n=0,\pm 1,\pm 2\ldots ).$$

**Figure 2:**
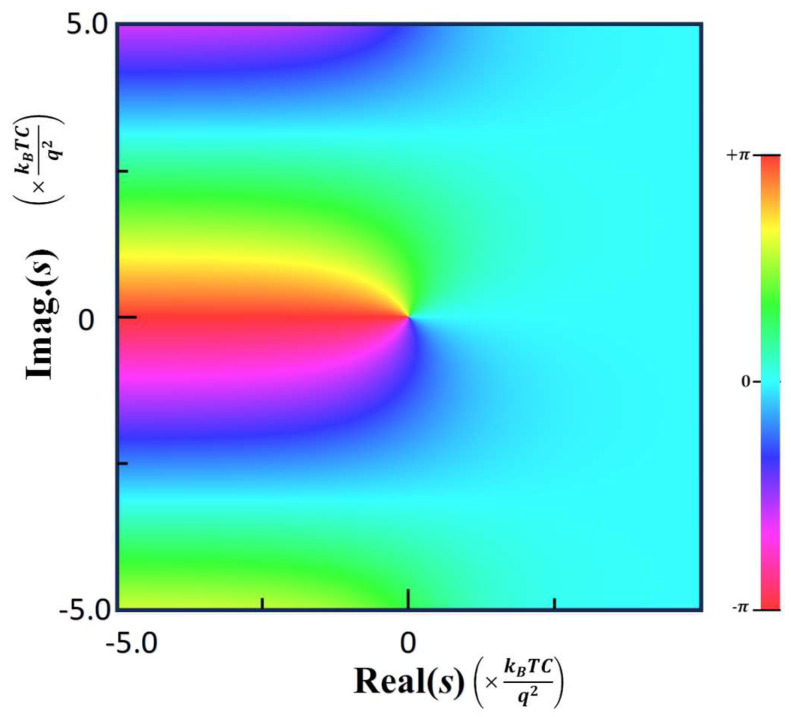
A phase map of the zeta function, $\zeta_{lo}\left(s\right)$, mapped in a complex plane showing a pole located on the imaginary axis.

Thus, all the poles are located on the imaginary axis as shown on a phase map of $\zeta_{lo}\left(s\right)$ calculated in Figure 2. It is noted that the phase rotates 2π around the poles, observed as the full color transition around the origin.

The characteristic behaviors of $\zeta_{lo}\left(s\right)$ are found along the imaginary axis (x = 0) and along or in parallel to the real axis (y = 2nπ/a) as follows. Substituting Equation (22) into Equation (20) and setting x = 0, we obtain $\zeta_{lo}\left(j\cdot y\right)$ along the imaginary axis as follows:

(24) $$\zeta_{lo}\left(j\cdot y\right)\;=\;\frac{1}{2}-\frac{j}{2}\cot\left(\frac{ya}{2}\right).$$

**Figure 3:**
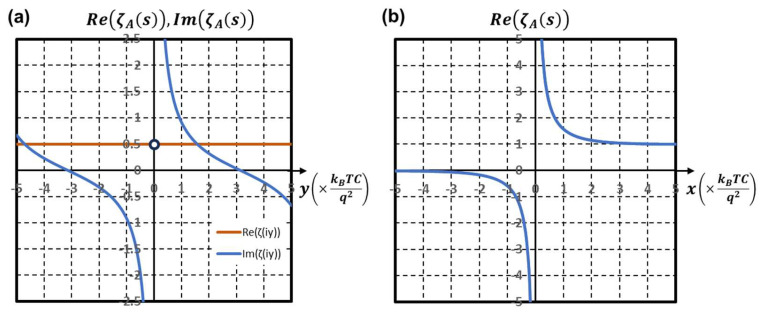
(**a**) Real (orange line) and imaginary (blue line) parts of $\zeta_{lo}\left(s\right)$ evaluated along the imaginary (y-) axis derived as (24). At x = 0, the real part is indeterminant, represented as a white circle. (**b**) The real part of $\zeta_{lo}\left(s\right)$ evaluated along the real (x-) axis derived as (25).

Thus, the real part of $\zeta_{lo}\left(j\cdot y\right)$, ${Re(\zeta }_{lo}\left(j\cdot y\right))$, is constant, ($=\frac{1}{2}$) except at poles. ${Re(\zeta }_{lo}\left(j\cdot y\right))$ and the imaginary part, ${Im(\zeta }_{lo}\left(j\cdot y\right))$, are plotted in Figure 3a. At poles, ${Re(\zeta }_{lo}\left(j\cdot y\right))$ is indeterminant. Such a property is found by setting y = 0 in (20) giving a real function, $\zeta_{lo}\left(x\right)$,

(25) $$\zeta_{lo}\left(x\right)=\frac{1}{1-\exp\left(-xa\right)},$$

along the real axis as plotted in Figure 3b. Near x = 0, $\zeta_{lo}\left(x\right)$ diverges positively or negatively according to the sign of x. This sign inversion is also observed in Figure 2 of the phase map as the color rotation around the origin. It shows the phase rotation of π after sign inversion along the horizontal axis.

It is noted that for a positive integer of *s = m*, $\zeta_{lo}\left(m\right)$ coincides with the *m*-th order cumulant, $Cum\left(m,\;k\right),$ Equation (11), in the k→∞ limit normalized by the order parameter as follows:

(26) $$\zeta_{lo}\left(m\right)\;=\;\frac{1}{1-\exp\left(-ma\right)}\;=\;\underset{k\to \infty }{\lim}Cum\left(m,\;k\right)/\left(m-1\right)!.$$

Thus, cumulants are regarded as special cases of the linear operator zeta function. Conversely, $\zeta_{lo}\left(s\right)$ may be represented by the analytic extension of cumulants as follows:

(27) $$\zeta_{lo}\left(s\right)\;=\;\underset{k\to \infty }{\lim}Cum\left(s,k\right)/\Gamma (s),$$

where Γ(s) is the Gamma function representing the analytic extension of $\left(m-1\right)!$ [25]. One could also define a zeta function, $\zeta_{lo,k}\left(s\right)$, with a finite value of *k* from the cumulant as

(28) $$\zeta_{lo,k}\left(s\right)=\frac{1-\exp\left(-k\cdot sa\right)}{1-\exp\left(-sa\right)}=Cum\left(s,k\right)/\Gamma (s).$$

To set up a functional equation, we construct a modified zeta function, $\tilde{\zeta_{lo}}\left(s\right)$, by multiplying Equation (20) by a factor exp(−sa/2),

(29) $$\tilde{\zeta_{lo}}\left(s\right)\;\equiv \;\frac{1}{1-\exp\left(-sa\right)}\cdot \exp\left(-\frac{sa}{2}\right)=(-1)\cdot \tilde{\zeta_{lo}}\left(-s\right).$$

The second equality expresses that $\tilde{\zeta_{lo}}\left(s\right)$ is an odd function.

### 3.3. The Euler-Product-Type Zeta Function

In $P_{k}\left(t\right)$, Equation (7), the coefficients of exponential-time-dependent terms, an alternating function of all $\lambda_{i}$’s, are the most characteristic form. The terms arise from the inverse Laplace transform of the MGF [16]. These coefficients are not considered as probabilities of some events due to possible sign reversal depending on the value of $\left({\lambda_{j}-\lambda }_{i}\right)$ [26]. However, interpretation based on time-integrated probability rate allowing both positive and negative contributions is possible as follows. First, from Equations (3) and (4) and the initial conditions, we obtain

(30) $$P_{0}\left(t\right)=\exp\left(-\lambda_{0}t\right),$$

(31) $$P_{1}\left(t\right)=\frac{\lambda_{0}}{\lambda_{1}-\lambda_{0}}\exp\left(-\lambda_{0}t\right)+\frac{\lambda_{0}}{\lambda_{0}-\lambda_{1}}\exp\left(-\lambda_{1}t\right).$$

Taking account of Equations (4), (30), and (31) leads to

(32) $$P_{1}\left(t\right)=\frac{dP_{0}\left(t\right)}{dt}\cdot \Delta_{t}+\lambda_{0}\exp\left(-\lambda_{1}t\right)\cdot \Delta_{t},$$

where $\Delta_{t}\;=\;1/\left(\lambda_{0}-\lambda_{1}\right),$ acting as an integration time or an effective lifetime with the effective emission rate of $\left(\lambda_{0}-\lambda_{1}\right)$. Thus, the first term is regarded as the “variation rate of $P_{0}\left(t\right)$ integrated over the period $\Delta_{t}$”. Similarly, the second term is interpreted as follows: $\lambda_{0}\exp\left(-\lambda_{1}t\right)$ describes an increasing rate of the probability of staying in the 1-st state ($=\exp\left(-\lambda_{1}t\right)$) due to the escape rate (${=\lambda }_{0}$) from the 0-th state integrated over $\Delta_{t}$. Similarly, a general term, $\frac{\lambda_{j}}{\lambda_{j}-\lambda_{i}}\exp\left(-\lambda_{i}t\right)$, describes “the variation rate of the *i*-th state probability due to variation in the j-th state probability integrated over the effective lifetime, $1/\left(\lambda_{j}-\lambda_{i}\right)$”. Thus, successive transitions described by Equation (3) lead to the product term representing the weight of the contribution from the other states to the *i*-th state.

Now, we investigate the coefficient of the *k*-th term of the PDF as a fundamental term. Using Equations (2) and (15), and by assuming $\lambda_{0}\;=\;1$, the expression is modified as follows:

(33) $$\Pi_{SR}\left(k\right)\;\equiv \;\prod_{i=1}^{k-1}\frac{1}{1-\frac{\lambda_{k}}{\lambda_{i}}}\;=\;\prod_{i=1}^{k-1}\frac{1}{1-\lambda_{i}}\;=\;\prod_{i=1}^{k-1}\frac{1}{1-{\tau_{i}}^{-1}}.$$

Equation (33) is formally identical to the Euler product, $\Pi_{p}\left(k\right)$, of the integers by replacing $\tau_{i}$ with $p_{i}$, the *i*-th prime number [19],

(34) $$\Pi_{p}\left(k\right)=\prod_{i=1}^{k-1}\frac{1}{1-{p_{i}}^{-1}}.$$

Because $\lambda_{i}\;=\;{\tau_{i}}^{-1}<1\;(i>0)$, $\Pi_{SR}\left(k\right)$ is a product of converging geometric series,

(35) $$\Pi_{SR}\left(k\right)=\prod_{i=1}^{k-1}\left(1+\lambda_{i}+{\lambda_{i}}^{2}+\ldots \right).$$

In the k→∞ limit, on expanding Equation (35), one finds any middle term in Equation (14). This parallels with the fact that any inverse of integer is generated by expanding Equation (34) in the k→∞ limit because of Equation (16) indicating the correspondence between $\tau_{i}$ and $p_{i}$.

By extending the exponent to a complex domain, *s*, and taking the k→∞ limit, we obtain a zeta function of the Euler product form as

(36) $$\zeta_{EP}\left(s\right)=\prod_{i=1}^{\infty }\frac{1}{1-{\tau_{i}}^{-s}}=\prod_{i=1}^{\infty }\frac{1}{1-\exp\left(-i\cdot sa\right)}.$$

The second equality is due to Equation (2), and a is defined by Equation (21). Using Equations (20) and (36), it can be expressed as a product of $\zeta_{lo}\left(i\cdot s\right)$’s,

(37) $$\zeta_{EP}\left(s\right)=\prod_{i=1}^{\infty }\zeta_{lo}\left(i\cdot s\right).$$

In the same way as $\zeta_{lo}\left(i\cdot s\right)$ is analyzed, we specify the locations of poles. By setting the denominator of Equation (36) to be zero, the poles are obtained as

(38) $$x=0,\;y=\frac{2n}{i}\pi \cdot \left(\frac{k_{B}TC}{q^{2}}\right),\;n=0,\pm 1,\pm 2,\;\ldots ,\;i=1,\;2,\ldots .$$

**Figure 4:**
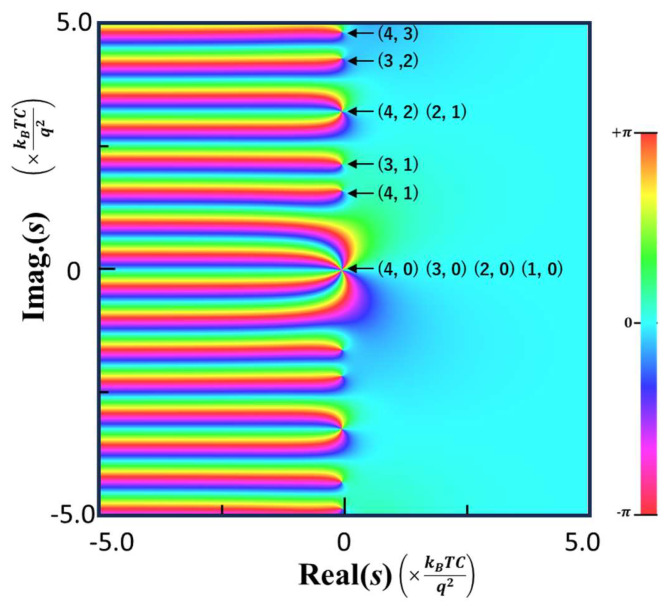
A phase map of the zeta function, $\zeta_{EP}\left(s\right)$, mapped in a complex plane showing poles located on the imaginary axis. The poles with *y* ≥ 0 are indicated by arrows together with the pairs of indices, (i,n), specified by Equation (38).

Thus, all the poles are located on the imaginary axis with shorter separations (divided by *i*) than those of $\zeta_{lo}\left(s\right)$. A calculated phase map of $\zeta_{EP}\left(s\right)$ with *i* = 4 is shown in Figure 4. The poles with *y* ≥ 0 are indicated by arrows on the right side together with the pairs of parameters, (i, n) specified by Equation (38). The number of the pairs corresponds to the order of the pole. For instance, the order of the pole at the origin is four, consistent with the observed four times full color transitions. In Appendix A, an alternative derivation of Equation (36) from a formal definition of the zeta function of a matrix [21] is outlined.

As for the functional equation, since we find $\tilde{\zeta_{lo}}\left(i\cdot s\right)$ is an odd function, Equations (29) and (37) are indeterminant in the limit k→∞. For a finite k value, defining $\tilde{\zeta_{EP,k}}\left(s\right)$ as

(39) $$\tilde{\zeta_{EP,k}}\left(s\right)\;\equiv \;\prod_{i=1}^{k}\zeta_{lo}\left(i\cdot s\right)\cdot \exp\left(-\frac{i\cdot sa}{2}\right),$$

the following functional equation dependent on k is obtained:

(40) $$\tilde{\zeta_{EP,k}}\left(-s\right)\;=\;{\left(-1\right)}^{k}\cdot \tilde{\zeta_{EP,k}}\left(s\right).$$

Finally, it is noted that $\zeta_{EP}\left(s\right)$, Equation (37), may be expressed by cumulants by formally substituting s for i·s in Equation (27),

(41) $$\zeta_{EP}\left(s\right)=\prod_{i=1}^{\infty }\left(\underset{k\to \infty }{\lim}Cum\left(i\cdot s,k\right)/\Gamma \left(i\cdot s\right)\right).$$

## 4. Discussion

### 4.1. Correspondence Between the Prime Number Problem and the Soft-Reset Statistics

The main results of the previous sections are summarized from the first to the fifth rows of Table 1. It is noted that the zeta functions of the soft-reset are found to be equivalent to the analytic extension of the cumulants of the PDF (4th row). As a special case of s = 2 (6th row), the soft-reset noise problem formally corresponds to the Basel problem, i.e., the infinite sum of the inverse square of the integers approaching $\frac{\pi^{2}}{6}$ [27].

Finally, the PNT states that [19,20], for a large integer n, the number of the primes not exceeding n, denoted commonly as π(n), is approximately given as

(42) $$\pi (n)\sim \frac{n}{ln(n)}.$$

The corresponding description in the soft-reset problem is described as follows: in a given “sufficiently long” period of time, *t*, so that *t* is approximated as $\tau_{k}$, and that the effect of $\lambda_{0}$ can be neglected or approximated as $\lambda_{0}\;$= 1, the number of the occurrences of the prime elements (periods) or of the emitted electrons during the period *t* is *k*. From Equations (2), (15), and (21),

(43) $$k\sim \ln\left(\tau_{k}\right)/a.$$

Therefore, the correspondence of the PNT is obtained as the average lifetime of one emission event, $\bar{\tau_{k}}$,

(44) $$\bar{\tau_{k}}\sim a\cdot \frac{\tau_{k}}{\ln\left(\tau_{k}\right)},$$

which has an identical form with Equation (42) apart from the scaling factor a, as listed at the bottom row of Table 1.

It is also noted that Equation (43) can be directly obtained from the solution of the diode equation, Equation (35) of Ref. [16], reproduced as

(45) $${V\prime }_{k}=\frac{kq}{C}=\frac{k_{B}T}{q}\cdot \left\{\ln\left(a<t_{k-1}>\right)+1\right\}.$$

With the approximation $<t_{k-1}>\sim \tau_{k}$ and $\ln\left(\tau_{k}\right)\gg 1,$ one obtains Equation (43). Therefore, the solution of the diode decay equation [28] is equivalent to the analog of the PNT. Conversely, the analog of the PNT, Equation (42), is reproduced by the measurable quantities of the SR-process, ${V\prime }_{k}$ and time. The significance of Equation (45) is that the time axis is factored into “countable prime elements ($\tau_{k}$)” holding the identical relation to the PNT Equation (42).

**Figure 5:**
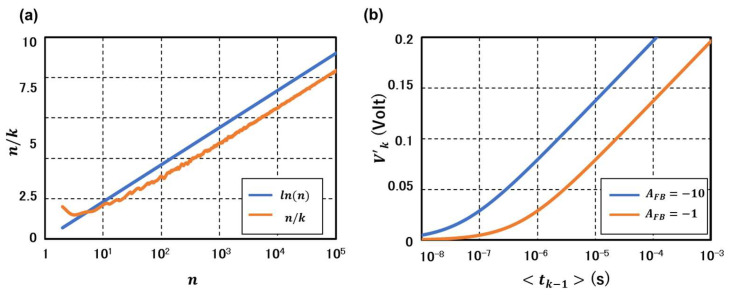
(**a**) Blue curve: a natural log plot of integers from 2 to 10^5^ representing *n*/*k* where *k* is the suffix of prime numbers. Orange curve: Plot of *n*/*k* obtained from the actual integers and prime numbers. (**b**) Time dependent ${V\prime }_{k}$ during a soft-reset calculated from (45). The orange curve is for the case of single-charge correlation only. The blue curve is calculated with an external negative feedback circuit.

In Figure 5a, curves of n/π(n) calculated from Equation (42) (blue) and from the actual integers and the prime numbers (orange) are plotted. The blue line is simply ln(n), representing the orange curve well except for the low n (<~20) range. In Figure 5b, a curve calculated from Equation (44) (orange) is plotted. For a long time region (≥~$10^{-6}$ s), a logarithmic dependence of the curve similar to those plotted in Figure 5a is observed as expected from Equation (44). In addition, the blue curve is obtained with an effective feedback gain of 10 or the parameter a replaced by 10a in Equation (44) [16]. Since the asymptotic regime starts from a much earlier time, ~$10^{-7}$ s, the larger feedback gain is confirmed to give a much shorter reset period, representing the effect of the feedback noise suppression [16]. It is also noted that the increase in $V_{k}$ at any time of the blue curve reflects the increased stored charge due to increased feedback gain.

### 4.2. Generality and Limitations of the Present Assumptions in Mesoscopic Devices

The appearance of the Euler product, Equation (33), is due to the non-degeneracy of the eigenvalues of the matrix operator A in Equation (5). The physical origin of the term is firstly ascribed to the Coulomb (correlation) interaction of a single charge with the surrounding dielectric medium represented by a capacitance (assumption (i) in Section 2.2). Then, the Markovian property (assumption (ii)) and the independence (assumption (iii)) of the emission process led to the difference-product form. Such a term would be a general description for charges in a weakly interacting regime where charges can be modeled only by Coulomb’s law [29]; any energy level or position is occupied only by a single charge at the same time. The corresponding charge concentration is typically 10^18^~10^20^ cm^−3^. These conditions are met by many mesoscopic devices, such as those based on two-dimensional electron gas (2DEG). A typical example is a wave function ($\Psi_{\upsilon }$) of the quantum Hall effect (QHE) consisting of a coefficient of a non-interacting part ($\Phi_{\upsilon *}$) multiplied by a difference-product of positions ($\prod_{j<k}\left(z_{j}-z_{k}\right)$) describing the interacting electrons [29],

(46) $$\Psi_{\upsilon }=\Phi_{\upsilon *}\cdot \prod_{j<k}{\left(z_{j}-z_{k}\right)}^{2p},$$

where 2p is the number of the magnetic flux quanta associated with a charge. It is noted that, apart from the degeneracy due to the flux quanta (2p), terms in the difference product are mutually prime, to which similar discussions as above could be applicable. Considering these common structures, the present analyses would be useful in specifying each charge or counting the number of charges in the Coulomb interacting charge systems.

Finally, we mention the limitations of the present analysis set by the condition specified in Section 2.1, i.e., $k_{B}T\gg q^{2}/C$. If the device scale were reduced to much smaller than the mesoscopic regime or less than a 10 nm scale, the effective single-charge energy would be much larger than the assumed one (≲150 mV), resulting in a barrier height increase with a much smaller number of emissions. In such cases, the approximation taken by the asymptotic regime, i.e., the k→∞ limit, is not valid. Physically, this means that the noise suppression or cumulants convergence effect would not be effective. Thus, the present analyses are indeed closely related to submicron-scale devices operated at RT.

## 5. Conclusions

The single-charge correlation effect during the SR is described as the cumulants of the governing PDF at room temperature. They are extended to a complex domain identified as the zeta functions. The three fundamental assumptions, i.e., single-charge correlation energy, the Markovian property of each charge emission event, and the independence of each state, lead to an equivalent circuit model (Figure 1c) and to the generalized moment function generated by the lifetimes of the states identified as the prime elements in Equation (14). Following the standard analyses in the prime number problem, two types of zeta functions are constructed and investigated. They are found to be analytic except for poles—Equations (20) and (36). Functional equations are found as Equations (29) and (40). Also, the zeta functions are found to be expressed by analytic extension of the cumulants—Equations (27), (28), and (41). The characteristic Euler products in the PDF are interpreted as the probability change rate integrated by an effective lifetime of each emission state—Equation (32). Finally, between $\tau_{k}$ and k, an analog of the PNT is found as Equations (43) and (44). Thus, the algebraic structure of the cumulants and the analytic properties of the associated zeta functions are found to be highly analogous to those of the prime number problem of the integers (Table 1).

## Figures and Tables

**Table 1:** Correspondence between prime numbers and the soft-reset.

Item	Prime Number Problem	Soft-Reset
Basic Ring	Integers: $\prod_{i=1}^{k}{p_{i}}^{n_{i}}$	Polynomials (Moments): $\alpha \prod_{i=0}^{k-1}{\tau_{i}}^{n_{i}}$
Prime Elements	Prime Numbers: $p_{i}$	Lifetimes (Eigen Values) $\tau_{i}(\equiv 1/\lambda_{i})$
Euler Product	∏i1−1pi−1	∏i1−1τi−1
Zeta Functions	$\zeta_{R}(s)$	$\zeta_{lo}\left(s\right)$, $\zeta_{EP}\left(s\right)$ ~$Cum\left(s,k\right)$
Poles/Zeros	Poles and zeros	Poles on the imaginary axis
ζ(2)	Basel problem $\sum \frac{1}{n^{2}}\;=\;\frac{\pi^{2}}{6}$	Soft-reset noise problem $\sum \frac{1}{{\tau_{i}}^{2}}\;=\;\frac{k_{B}TC}{2q^{2}}$
Prime Number Theorem	$\sim \frac{n}{ln(n)}$	$\sim a\cdot \frac{\tau_{k}}{ln(\tau_{k})}$

## Data Availability

The data presented in this study are not available due to privacy concerns.

## Abbreviations

The following abbreviations are used in this manuscript:

SR	Soft-Reset
PDF	Probability Distribution Function
HDF	Hypoexponential Distribution Function
KB	Kolmogorov–Bateman
PNT	Prime Number Theorem
FD	Floating Diffusion
MGF	Moment Generating Function
SET	Single Electron Transistor

## Appendix A. Derivation of $\zeta_{EP}\left(s\right)$ from Its Definition

$\zeta_{EP}\left(s\right)$, Equation (36), can be derived from its definition as follows. The zeta function of a matrix A, $\zeta_{A}\left(s\right)$, is defined as [21],

(A1) $$\zeta_{A}\left(s\right)=\frac{1}{det(I+A\cdot \exp\left(-s\right))}\;,.$$

where I is an identity matrix and A is given in Equation (5). Using the diagonalization matrix, U and $U^{-1}$, given as (A5) and (A9) in Ref. [16], respectively,

(A2) $$\zeta_{A}\left(s\right)=\frac{1}{\det U^{-1}\left(I+A\cdot \exp\left(-s\right)\right)U}=\frac{1}{\det\left(I+U^{-1}AU\cdot \exp\left(-s\right)\right)}.$$

Because $\left(I+U^{-1}AU\cdot \exp\left(-s\right)\right)$ is a diagonal matrix with the *j*-th column (row) element of $\left(1-\lambda_{j}\cdot \exp\left(-s\right)\right)$, $\zeta_{A}\left(s\right)$ is obtained as

(A3) $$\zeta_{A}\left(s\right)=\prod_{j=1}^{\infty }\frac{1}{1-\lambda_{j}\cdot \exp\left(-s\right)}=\prod_{j=1}^{\infty }\frac{1}{1-\exp\left(-ja-s\right)}.$$

For the second equality in (A3), relation Equation (2) is used. By mapping the variable from s to s′, such that −ja−s = −ja·s′ is satisfied, $\zeta_{A}\left(s\right)$ is also mapped to $\zeta_{EP}\left(s\prime \right)$ as

(A4) $$\zeta_{EP}\left(s\prime \right)=\prod_{j=1}^{\infty }\frac{1}{1-\exp\left(-ja\cdot s\prime \right)}.$$

Rewriting s′ as s, we obtain $\zeta_{EP}\left(s\right)$ as Equation (36).
